# Bugs in the Virtual Clinic: Confronting Telemedicine’s Challenges Through Empathy and Support

**DOI:** 10.2196/25688

**Published:** 2022-04-22

**Authors:** Bradley H Crotty, Melek Somai

**Affiliations:** 1 Inception Labs Collaborative for Healthcare Delivery Science Medical College of Wisconsin Milwaukee, WI United States

**Keywords:** telemedicine, virtual care, patient experience, consumer informatics, telehealth, access, challenge, electronic health record, digital health

## Abstract

Although telemedicine has been an important conduit for clinical care during the COVID-19 pandemic, not all patients have been able to meaningfully participate in this mode of health care provision. Challenges with accessing telemedicine using consumer technology can interfere with the ability of patients and clinicians to meaningfully connect and lead to significant investments in time by clinicians and their staff. In this narrative case, we identify issues related to patients’ use of technology, make comparisons between telehealth adoption and the deployment of electronic health records, and propose that building intuitive and supported digital care experiences for patients is required to make virtual care sustainable.

## Introduction

### Background

“It’s okay… just tell me what you see on your screen.” A deep sigh punctuated with frustration was clearly audible across the phone line. It was a little after 6 PM. With the clinic staff having left an hour ago to begin their weekends, Ms J continued to struggle with her doctor to get connected to our telemedicine system for a virtual examination.

As informaticists who lead our health system’s digital health program, we work to scale up virtual care capabilities and make them quickly accessible for patients and clinicians, who are all rapidly adapting to new challenges. Through experiences described here, we reflect on patient adoption of technology as a medium for care, draw similarities between the history of electronic health record (EHR) adoption and virtual care adoption, and contemplate how building meaningful digital care experiences for patients rather than creating digital carbon copies of traditional care is the way forward.

Ms J, a gregarious woman in her 70s with mild chronic obstructive pulmonary disease and well-controlled hypertension, had been scheduled for a telephone check-in earlier in the day. It was during the initial COVID-19 wave, when personal protective equipment was scarce and hospitals, including ours, were managing most patients virtually for the first time. The front desk staff had left a comment in the schedule: “Patient cannot do a video visit.” As clinical informaticians, we had been struck by how fast health care transitioned to using video as the primary means for ambulatory care during the COVID-19 pandemic. And although our practice had been working to increase the number of video visits, clinicians defaulted to phone calls to provide care for those unable to access video visits, such as Ms J. As it turned out, Ms J’s complaint over the phone was a new one, painful bumps on her skin “like bug bites but more purple,” a problem for which at least a virtual exam would be warranted to begin the diagnostic process. Compared to a phone call, a video visit can allow for important nonverbal communication, further aiding the diagnostic and therapeutic process, such as the ability to confirm that both the patient and the clinician understand the information being shared through facial expressions [1,2]. Without video, the differential diagnosis was too broad, ranging from bug bites to palpable purpura.

In the COVID-19 era, the need for physical distancing, the shortages of personal protective equipment, and the patchwork of state-issued guidance around stay-at-home orders had driven patients and providers alike to flock to virtual care. Insurance companies and the Centers for Medicare and Medicaid Services had also agreed to cover many telemedicine services, a step the federal government has committed to maintaining, including flexibility for audio-only visits for Medicaid beneficiaries [3,4]. With clear incentives for its use and the regulatory and reimbursement limitations historic to telemedicine dissolved at once, virtual care had now spread broadly across the country [5]. This represented the first real growth of telemedicine using consumer technology. Within our system, ambulatory care went from 0.25% to 70% virtual within 2 weeks of the pandemic. But as we see virtual care receding, locally accounting for 10% of all ambulatory care, now is the time to focus on creating sustainable virtual experiences following this large-scale “experiment.” We foresee a hybrid care model being adopted moving forward, meaning that clinical offices will intermix in-person and virtual care within the same clinical sessions. Surveys have shown that 70% to 75% of people plan to use some form of virtual care moving forward [6,7]. Analyses of outpatient claims data have also revealed that 20% of care could reasonably be virtualized. To sustain virtual care as we transition to a hybrid care model, we must reflect on the experiences of patients and clinicians in adopting virtual care technology, incorporate lessons from the digitization of health records, and identify ways to deliver intuitive and supported experiences.

### Technology Adoption

Reflecting on recent clinic schedules, we wonder how the future of medicine can become digital by default. Although the uptake of virtual care before the COVID-19 pandemic had been modest, generally used by those comfortable with technology or seeking convenience, Inception Health, our innovation lab within the Froedtert & the Medical College of Wisconsin health network, had been laying the groundwork for broader digital health adoption. Reducing the complexity, cost, and hassle of health care were the goals, digital technology was the medium, and consumerism and competition were the levers. The first patient that either of us ever took care of through video-based care was a man in his 40s who worked in the information technology industry. He became a patient after presenting with diabetic ketoacidosis as an adult. After his diagnosis, he had diligently watched his diet and digitally tracked his blood glucose and he eventually stopped receiving any insulin-based therapy. He was an example of, as Everett Rogers, who framed the diffusion of innovation curve, would say, an “early adopter” [8]. He had comfort with the medium and the financial resources and capabilities to easily connect with a clinician. The COVID-19 pandemic and the limitations of in-person care have pulled Rogers’ diffusion of innovation curve leftward, dragging most patients and providers into a new mode of care. We were trying to get Ms J there, too.

And here lies one of the biggest challenges for digital care. Unless our profession achieves equal access and experiences for all our patients, digital and video visits may amplify the divide in care quality between those able to easily access care and those who face major barriers: older or differently abled populations, underserved populations, and racial and ethnic minority populations [9]. Data have shown that up to 38% of older patients are unprepared for telemedicine [10]. Over one-quarter of Medicare beneficiaries lack any internet access altogether, an issue that disproportionately affects communities of color and patients with lower educational attainment [11]. Our experiences during the COVID-19 pandemic highlighted that minority patients were less likely to schedule video visits than telephone visits [12]. Furthermore, when patients did schedule video visits, we witnessed our older and more socioeconomically disadvantaged patients having lower success rates in connecting with their physicians [13].

### Echoes of Electronic Health Record Adoption

As health care digital transformation is underway, much can be learned from the impact, pitfalls, and challenges of implementing EHR systems in the past decades that can inform us about the road ahead. As clinicians, we are already concerned that the EHR has turned us into data entry clerks, commandeering the practice of medicine and leading to “death by a thousand clicks” [14]. In parallel, this revolution of virtual care has begun to turn all medical staff into legions of technical supporters, adding to the list of required skills for certain jobs and the time required for visits. Pressing the “connect” button with each visit brings uncertainty. Will this work? Will we see and hear them, and will they see and hear us? A small amount of dopamine bursts when the patient’s image appears, coinciding with a sigh of relief.

For many patients, their telemedicine visits are their first virtual care experiences, and for many older physicians, EHRs may have been their first intensive computing experience. To connect to their clinicians, patients navigate instructions, download apps, and check themselves in, oftentimes with little assistance and using a mere 6-inch screen; they could experience difficulties at any step along the way. Ms J downloaded the required app, but she was still working through the check-in screens: “It says e-check-in, medications, allergies… but I don’t see what it wants me to do next.” Ms J had worked with our new digital support center after our initial discussion to ensure she installed the right mobile app and to log into her account, which required her to confirm her email account and set up a strong password with at least one special character. Our digital check-in configuration required our patients to verify their insurance, medications, and electronically sign any required documents, mirroring steps that staff usually take during in-person visits. The unfamiliar user interface, one that required scrolling to read small print on a mobile device, was difficult to overcome, and the user’s frustration was mounting. Despite our digital support center, medical assistants, and adaptation to the new role of digital physicians, we had failed to connect to the patient by video, a fate that became a pattern of a failure of digital care as a whole. In data from our organization’s experience, 1 in 10 scheduled video visits shifted to telephone visits [13].

As health care shifts to digital platforms, clinicians must learn from and avoid the mistakes we made during our attempts to digitize medical records. Merely digitizing documents, adapting paper forms into electronic copies, accomplishes only a fraction of digital capability. Digital technology provides power by automating routine tasks, providing improved reliability and availability, and creating new possibilities. Although EHR implementations were a prerequisite for digital care, they created their own problems: they have been associated with increasing clinicians’ workloads and physician burnout [15], were not easily interoperable with one another [16], contributed to increasing health care costs [17], became more complex over time [18], and were linked with a reduction in some elements of patient-physician communication [19]. A modern synopsis of EHRs indicates that they are often “feature rich, yet function poor” [20]. We may very likely amplify those challenges and pitfalls if they are not considered carefully as we transition to digital and virtual care services for patients.

### Creating Better Experiences

To sustainably move forward, experiences like Ms J’s, and many others, have taught us that we must take different approaches and consider technology as part of the fabric of care rather than a mere medium. At our organization, we have embarked on a reengineering and rebuild of our virtual care experience. Rather than trying to substitute video visits for in-person care, we should conceptualize how the continuum of the care experience can be redesigned, combining the advantages of virtual and in-person care, the power of computing with human empathy, and a seamless digital pathway with timely access to the care team. The following questions should be considered: Which patients scheduled for the day could be seen through quick digital or telephonic check-ins? How can we use digital technology to rightsize the attention needed rather than providing patients with a standard visit length?

The following 2 principles guide our approach for creating sustainable virtual experiences: (1) intuitive experiences, which aim to make digital care easily accessible across the technological literacy continuum without the need for extensive training and (2) digital navigation and support, which aims to reimagine support for patients as they navigate digitally native health care interactions.

#### Intuitive Experiences

Drs Warner Slack and Howard Bleich, 2 pioneering physician-informaticians, both often quipped that “the quality of the computing is inversely proportional to the thickness of the training material, or length of training” [21]. We must work toward building accessible experiences for patients that incorporate empathy for users and examine all steps along the user journey. This requires taking a 360-degree view of the end-to-end experience [22]. Steps to reduce the cognitive load experienced by users and create simple interfaces that focus on a single step or task at a time with minimal scrolling required may help [23]. At a minimum, we should start by questioning each step in the virtual visit chain, from app download to electronic check-in, that a patient is asked to complete: Is this truly required for clinical care, and how else may we accomplish it [24]?

Embracing agile principles, which promote outcomes rather than processes, may help sustain this flexibility and develop better ways of improving the value of the experience for patients [25]. Working closely and collaboratively with patients as users of the tools and using frameworks like human-centered design with frequent user-testing, can identify design flaws early and help create more intuitive experiences.

#### Digital Navigation and Support

The goodwill of clinicians to provide technical support, as one of us was doing that Friday evening, is a very scarce resource. Clinicians must take care of people, not their technology, or so we believe. “The tech just needs to work,” a colleague told us. And yet, technology is far from the only issue. In clinical informatics, we often use the sociotechnical theory to guide technology implementations, which requires an understanding of people, processes, human-computer interactions, technology, and the interdependencies of these components [26]. Our implementation followed these tenets, but perhaps from the perspective of early adopters, not late adopters. Multiple layers of support are likely needed, such as having clinical staff who are knowledgeable about common issues, navigation support built into the digital experience, and family or community support for the technology.

Technical support may also become part of the care team, similar to how health coaches focus on assisting patients with behavior change or social workers support other needs such as transportation. A central competency of technology will be required across the caring professions; medical assistant training, for example, may have basic digital health technology as required learning. This know-how will also be crucial for encouraging patients to adopt other digital tools, such as digital therapeutics, that add evidence-based support in the form of apps or software [27].

In the hallways of our hospitals, staff with bright red blazers greet patients as they enter and help them navigate the hospital to reach their clinic appointments. What is the equivalent of such an experience in the digital realm? It may take new and different forms that health care systems must be willing to try. Perhaps avatars or bots may help patients prepare for visits, with the ability to connect with a support person if a patient has difficulty accessing their doctor. A successful digital support structure might become a standard in all health care systems rather than an afterthought.

For people who need more “at the elbow” support, or who lack internet connectivity or the ability to use technology, more instrumental support may be required. Partnerships with other organizations, such as those that already provide technical support as their core business, may also emerge to better support patients. For example, centralized support stations that can assist patients with telemedicine visits may open, either in satellite clinics, like more traditional telemedicine originating sites, or in community locations, such as pharmacies or senior housing.

What we have seen during the COVID-19 pandemic is that traditional ambulatory care in brick-and-mortar facilities plummeted, and virtual care was the primary option for nonemergent issues [28]. Virtual care was our main form of care; over the span of a few weeks, its users grew from early adopters to nearly everyone else. And although we saw challenges, such as those outlined, we also saw heroic achievements. The patient who had a virtual visit during a smoke break to discuss quitting, the immunocompromised patient who was worried about a COVID-19 rash (it was shingles), and the patient with unstable transportation who was able to see us the same day all come to mind as incredible victories. At a systems level, we must do all that we can to focus on the care, not the medium, to heal and care for our communities.

As for Ms J, she scheduled an in-person visit after the weekend. She was able to send in a picture of her foot using her phone camera, a consolation victory for the effort in connecting her to our portal, allowing us to triage her care. At her visit, it became clear, aided by the ability to attentively listen and observe, that her foot pain and the bumps were unrelated matters, and a diagnosis of plantar fasciitis and incidental varicosities was made, rather than a bug or spider bite. The only “bug” in the clinic was the difficulty in navigating the digital process.

Based on this lesson and others like it, our organization has implemented additional video tools to enable failover, circumventing barriers of portal sign-in and check-in requirements and adding more resources to our digital support arsenal. More broadly, we are scrutinizing every facet of the virtual care journey. We imagine others are, or should, be doing so as well.

## Abbreviations

EHR: electronic health record
